# Is Pediatric Melanoma Really That Different from Adult Melanoma? A Multicenter Epidemiological, Clinical and Dermoscopic Study

**DOI:** 10.3390/cancers15061835

**Published:** 2023-03-18

**Authors:** Vincenzo De Giorgi, Elisabetta Magnaterra, Biancamaria Zuccaro, Serena Magi, Manfredi Magliulo, Matelda Medri, Laura Mazzoni, Federico Venturi, Flavia Silvestri, Gian Marco Tomassini, Massimo Gola, Marta Tramontana, Samantha Berti, Ignazio Stanganelli, Luca Stingeni, Piero Covarelli

**Affiliations:** 1 Section of Dermatology, Department of Health Sciences, University of Florence, 50121 Florence, Italy; 2 Cancer Research “Attilia Pofferi” Foundation, 51100 Pistoia, Italy; 3 Skin Cancer Unit, Istituto Scientifico Romagnolo per lo Studio dei Tumori “Dino Amadori” (IRST) IRCCS, 47014 Meldola, Italy; 4 Dermatology Section, Department of Medicine and Surgery, University of Perugia, 06156 Perugia, Italy; 5 Dermatology Unit, Department Medicine and Surgery, University of Parma, 43121 Parma, Italy; 6 Surgical Oncology Section, Department of Medicine and Surgery, University of Perugia, 06156 Perugia, Italy

**Keywords:** melanoma, dermoscopy, estrogen, children, skin cancer

## Abstract

**Simple Summary:**

The salient clinical and epidemiological features, dermoscopic findings, and the clinical course of pediatric melanoma have not been well identified. Currently, the management of pediatric melanoma is based on adult guidelines, as specific treatment recommendations for children are unavailable. The most common dermoscopic feature of pediatric melanoma is the presence of irregular streaks/pseudopods (74.36%). When evaluating the total number of different dermoscopy criteria per lesion, 64.1% of the lesion assessments recognized two dermoscopic characteristics, 20.5% identified three, and 15.4% of the assessments documented four or more. All the pediatric melanomas analyzed presented at least two dermoscopic criteria of melanoma, suggesting that this could be a key for the dermoscopic diagnosis of suspected pediatric melanoma and making it possible to reach an early diagnosis even in this age group.

**Abstract:**

Purpose: To improve the diagnostic accuracy and optimal management of pediatric melanomas. Methods: We conducted a retrospective descriptive, multicenter study of the epidemiological, clinical, and dermoscopic characteristics of histopathologically proven melanomas diagnosed in patients less than 18 years old. Data on sociodemographic variables, clinical and dermoscopic characteristics, histopathology, local extension, therapy and follow-up, lymph node staging, and outcome were collected from the databases of three Italian dermatology units. We performed a clinical evaluation of the morphological characteristics of each assessed melanoma, using both classic ABCDE criteria and the modified ABCDE algorithm for pediatric melanoma to evaluate which of the two algorithms best suited our series. Results: The study population consisted of 39 patients with a histologically confirmed diagnosis of pediatric melanoma. Comparing classic ABCDE criteria with the modified ABCDE algorithm for pediatric melanomas, the modified pediatric ABCDE algorithm was less sensitive than the conventional criteria. Dermoscopically, the most frequent finding was the presence of irregular streaks/pseudopods (74.4%). When evaluating the total number of different suspicious dermoscopy criteria per lesion, 64.1% of the lesion assessments recognized two dermoscopic characteristics, 20.5% identified three, and 15.4% documented four or more assessments. Conclusions: Contrary to what has always been described in the literature, from a clinical point of view, about 95% of our cases presented in a pigmented and non-amelanotic form, and these data must be underlined in the various prevention campaigns where pediatric melanoma is currently associated with a more frequently amelanotic form. All the pediatric melanomas analyzed presented at least two dermoscopic criteria of melanoma, suggesting that this could be a key for the dermoscopic diagnosis of suspected pediatric melanoma, making it possible to reach an early diagnosis even in this age group.

## 1. Introduction

Cutaneous melanoma (CM) is one of the most dangerous and therapy-resistant varieties of human cancer. In Europe, the incidence is currently around 10 to 25 cases per 100,000 inhabitants [1]. The average age at diagnosis is 65 years, and it is rarely reported in children, occurring before the age of 18 years in less than 1% of cases and accounting for about 1 to 3% of all pediatric cancers [2,3]. Pediatric melanoma (PM) is defined as a melanoma that occurs during a child’s growth before the age of 18 or 21 years, according to the cut-off used for defining adulthood. Despite occurring so rarely, CM is the most common pediatric malignant skin cancer. The presence of giant congenital melanocytic nevus, a first-degree relative affected by the disease, mutations in CDKN2A or CDK4 genes, dysplastic nevus syndrome, neurocutaneous melanosis, DNA repair defects (such as xeroderma pigmentosum), and immunosuppression are the major risk factor for the onset of this disease [4]. Moreover, some studies report a higher frequency of PM among Hispanic children and adolescents compared with non-Hispanic whites [5,6].

Differences between childhood and adult melanoma have not yet been characterized, as the literature on this topic is sparse. Salient clinical and epidemiological features, dermoscopic findings, and the clinical course of the disease have not been well identified. Furthermore, the management of pediatric melanoma is currently based on the adult guidelines, as specific treatment recommendations for children are unavailable.

Histopathologically, pediatric-onset melanomas are classified into three categories: Spitzoid melanoma, melanoma arising in congenital melanocytic nevi, and conventional melanoma (term that refers to pediatric cases that on the basis of histological examination alone are indistinguishable from those of adults) [4,5].

The frequency of each of these three subtypes varies according to the age of the patients, with a higher prevalence of spitzoid melanoma in subjects younger than 12 years of age, while melanoma arising in congenital melanocytic nevi and conventional melanoma would be more frequent in teenagers [4,5]. Superficial spreading and nodular melanoma are the most frequent histological types among conventional melanoma cases, with a higher prevalence of nodular melanoma cases than in adults [3,4,5].

While rare histological types would appear to be more frequent, lentigo maligna is extremely rare among pediatric patients and has been reported only in patients affected by xeroderma pigmentosum [7,8].

According to small studies and reports, hormone production may also play a key role in onset and progression of PM cases. Several differences, not only histological, have been found between pre- and post-puberty cases. Furthermore, most pediatric cases are found among adolescent patients, and after puberty, PM’s incidence rate rises rapidly, particularly among teenage girls.

Moreover, the same studies indicate that the clinical, histological, and dermoscopic features of these tumors in adolescents are more similar to those of adult patients than those of young children [3]. Indeed, the 40% of melanomas with onset after age 10 do not meet the ABCDE criteria (Asymmetry, Border Irregularity, Color variegation, Diameter > 6 mm, and Evolution), in contrast to the 60% of those arising before the same age[5,9]. Precisely for this reason, new ABCDE criteria have been proposed in addition to the traditional ones in order to facilitate diagnosis and avoid misdiagnosis and delays in seeking care. These criteria include amelanotic, bleeding or bump, color uniformity, de novo, any diameter, and evolution [9].

According to the existing literature, PMs appear to be more frequently amelanotic and have regular margins. They also seem to be more frequently clinically nodular, surmounted by a bloody ulcer, and of variable dimensions, often less than the canonical 6 mm of adults. Particularly, a less recent study has highlighted that about a half of PMs collected were raised and amelanotic, simulating pyogenic granulomas, which is a very common diagnosis among children [9,10].

However, studies published on this topic are generally small and lack the statistical power to reach relevant conclusions. Furthermore, almost nothing has been written about the dermoscopic characteristics of PMs and how these features differ from those of adulthood. Dermoscopy is totally acceptable in children, as it is noninvasive. The most advanced and latest-generation dermoscopes can even be utilized without contact, completely avoiding any physical discomfort or emotional stress in little patients. The discovery of specific patterns may increase the diagnostic accuracy, improve the differential diagnosis, and expedite the initiation of therapies. This is an important point, as PMs have a more aggressive clinical course, presenting with thicker primary lesions and a higher incidence of sentinel lymph node metastases [5,9,11,12]. Clearly, this is due not only to the increased aggressiveness of these lesions in the pediatric population but also to diagnostic delay.

Wide local excision of the primary tumor with adequate margins (based on the overall tumor depth) is the standard of care. Currently, surgical margins are based on the guidelines for adults, although some authors support the need to reduce them in younger patients given the frequent anatomical or functional limitations due to smaller body surface areas [13].

To improve the diagnostic accuracy and optimal management of PMs, we performed a retrospective study of a large series of patients with PMs retrieved from the database of three dermatology units in Italy (Florence, Romagna, and Perugia) from 1995 to 2021. We assessed the epidemiological characteristics, clinical and dermoscopic features of the tumors and the outcomes of the patients.

## 2. Material and Methods

We conducted a retrospective descriptive, multicenter study of the epidemiological clinical and dermoscopic characteristics of histopathologically proven melanomas diagnosed in patients under 18 years of age. Data on the sociodemographic variables; clinical and dermoscopic characteristics; histopathology; local extension; therapy and follow-up; lymph node staging (clinical involvement, sentinel lymph node biopsy (SLNB), and complete lymph node dissection (CLND)); and outcomes were collected from the databases of three Italian dermatology units (Firenze, Romagna, and Perugia). All the patients included in the study were independently diagnosed. Given their young age, they were not subjected to periodic screening. They presented to our outpatient clinic for the onset of a new, rapidly growing melanocytic lesion. As far as we know, none of the PM cases we collected belonged to families involved by predisposing genetic mutations. According to the protocols in force in each center, each lesion was photographed clinically and dermoscopically before surgical excision of the primary melanoma. 

Patients and the legal guardians (when still needed) were informed about the study, and written informed consent for publication of the photographs used in this manuscriptwas obtained.

PMs histopathologically diagnosed between 1995 and 2021 were eligible for the current analysis. All lesions were examined by dermatopathologists specializing in the diagnosis of melanocytic skin tumors [14]. Since melanoma in prepubertal children may act differently than that in post-pubertal children, we decided to divide patients by age as a surrogate for pubertal status [15,16]. We divided patients into two age groups: group A, including patients aged 12 years and younger, and group B, including patients aged 13 to 18 years.

The equipment used for the dermoscopic examinations consisted of a handheld dermatoscope (Heine Delta 20, Heine Optotechnick, Herrsching, Germany). Both clinical and dermoscopic images of all lesions were captured with a high-resolution compact digital photographic camera (Olympus Digital model no. E-520, a 7.1 megapixel digital photo camera with a 3.8 optical zoom lens, a focal length of 28 to 105 mm in a 35 mm format, and a maximum lens aperture of f/2.8–f/5.8).

Dermoscopic images were captured via Dermaphot (Heine Optotechnick, Herrsching, Germany), which connects the dermatoscope to the camera to generate reproducible, high-quality dermoscopic pictures at 10-fold magnification in a joint photographic expert group (JPEG) file format. These clinical and dermoscopic images and the data were stored on a common Windows-based personal computer. 

We performed a clinical evaluation of the morphological characteristics of each assessed MM using both classic ABCDE criteria and the modified ABCDE algorithm for PM^9^ in order to evaluate which of the two algorithms best suited our series. 

Three investigators (GMT, IS, and VDG) with expertise in pigmented lesions and dermoscopy, who were blinded to the clinical history and final diagnosis of the lesion, analyzed the archived digital dermoscopic images, and completed a printed questionnaire to categorize the lesions according to a typical dermoscopic pattern analysis. These dermatologists possessed identical levels of training and experience in dermatology, each having over five years of practice in dermoscopy. The dermoscopic pattern and the presence or absence of dermoscopic features in a given lesion were defined by the agreement of at least two of the three dermoscopists. Descriptive analyses were performed to summarize the number and proportion of patients by demographics, tumor characteristics, clinical management, and outcomes.

## 3. Results

The study population consisted of 39 patients with a histologically confirmed diagnosis of PM, including 24 females (61.5%) and 15 males (38.5%). Their ages at presentation ranged from 5 to 18 years (mean 14.58 years, median 15 years, SD 3.38), with the majority of diagnoses (79.8%) during their teenage years (ages 13 to 18) (Table 1).

Remarkably, 10 out of 39 patients (25.6%) had a family history of melanoma, and 3 out of 39 (9.5%) were diagnosed with a second primary melanoma during follow-up. All patients were Caucasian. Melanoma in situ was reported in six girls and three boys; no in situ cases were diagnosed before the age of 14. Regarding invasive melanomas, the mean Breslow thickness was 1.05 mm (SD 0.23), with a striking difference between the two age groups (2.02 mm in the 0 to 12 years age group vs. 0.77 mm in adolescence). Notably, girls were mostly responsible for this difference; in fact, a significant decrease in Breslow thickness can be observed in the over 13 age group (falling from 2.36 mm in the 0 to 12 age group to 0.77 mm). Concerning histological type, superficial spreading melanoma was found in 20 cases (51.3%), spitzoid melanoma in 15 cases (38.5%), and acral lentiginous melanoma in 1 case (2.6%). However, in our retrospective series, we were not able to differentiate with certainty Spitzoid melanomas from Spitz melanomas/malignant Spitz tumors, according to the current WHO classification (4th ed.) due to lack of comprehensive molecular–genetic characterizations that, by the time of the original diagnosis, was not performed [17]. In two cases, superficial spreading melanoma had arisen in the context of a small congenital nevus. We did not find any nodular melanoma or any lentigo maligna in our series. Three rare melanomas were histopathologically recognized (nevoid melanoma, spindle cell melanoma, and deep penetrating melanoma).

The anatomical sites were as follows: trunk (39.5%), lower extremities (36.8%), upper extremities (15.8%), and head and neck (7.9%). Considering the whole study population, only one patient had distant metastasis and died from the disease. The mean follow-up period was 99 months (median 76 months, range 12 to 241 months). Among our patients, nine underwent SLNB, with evidence of metastases in four cases (44.4%).

**Figure 1:**
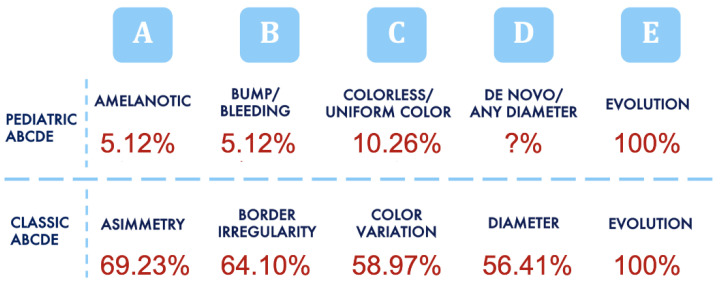
Comparison between pediatric and adult ABCDE algorithms. The classic ABCDE algorithm was the one that best suited our cases (69.2% of lesions were asymmetric, edges were irregular in 64.1% of cases, 59.0% of our PM cases presented clinically with two or more colors, and 56.4% were over 6 mm in diameter).

**Figure 2:**
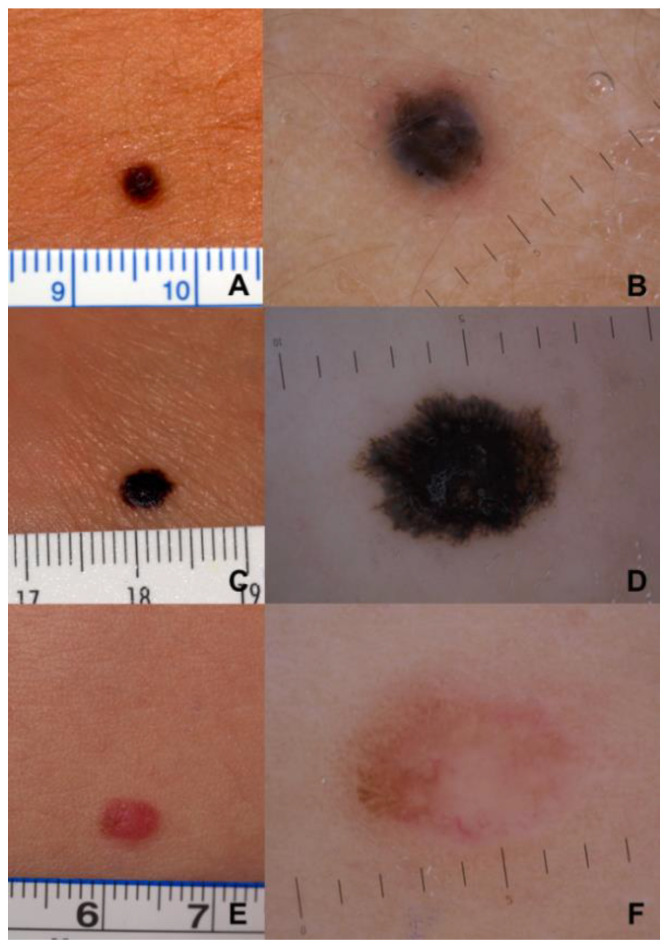
Clinical and dermoscopic images of PM (rulers are in millimeters). (**A**,**B**) Deep penetrating melanoma (1.9 mm Breslow thickness) of the back in a nine-year-old boy: (**A**) Clinical presentation. (**B**) Dermoscopy reveals irregular streaks and a blue–white veil. (**C**,**D**) Reed-like spindle cell melanoma of the left ankle in situ in an 18-year-old female: (**C**) Clinical presentation. (**D**) Dermoscopy reveals a multicomponent pattern with inverse network and irregular streaks/pseudopods. (**E**,**F**) Hypomelanotic nevoid melanoma (1.2 mm Breslow thickness) of the back on a 14-year-old girl: (**E**) Clinical presentation. (**F**) Dermoscopy reveals a pinkish background and an atypical vascular pattern with polymorphic vessels. In the superior part of the lesion, the atypical pigment network is detectable.

Clinically, 94.9% of the lesions were pigmented, and the remaining 5.1% of the lesions were achromic. The mean lesion size was 8 mm (median 6.8 mm). Comparing classic ABCDE criteria with the modified ABCDE algorithm for PMs (Figure 1), we found that, according to conventional ABCDE, the lesions were asymmetric and edges were irregular in 69.2% and in 64.1% of the cases, respectively. PMs presented clinically with two or more colors in 59.0% of our patients, and 56.4% were over 6 mm in diameter. Instead, pediatric ABCDE criteria were lacking in both age groups: features such as amelanotic, lesional bleeding, raised nodular lesions, and color uniformity were rare. The PMs were amelanotic in 5.1% of cases, as well as those presenting as bumps or nodules, and only 10.3% were a single color. Lesional evolution, a common feature of both algorithms, was nearly universal in both age groups (Figure 2A,C,E). Therefore, the modified pediatric ABCDE algorithm was less sensitive than the conventional one.

Dermoscopically, the most frequent finding was the presence of irregular streaks/pseudopods (74.4%) (Figure 2B,D). Atypical pigment networks, atypical globules, and regression structures were reported in 30.8% of the cases. In terms of vascular structures, an atypical vascular pattern was observed in 20.5% of the cases. Other detected dermoscopic features were blue–white veils (15.4%), an inverse network (10.3%), scar-like areas (10.3%), and a prominent network (10.3%)( Table 2). When evaluating the total number of different dermoscopy criteria per lesion, 64.1% of the lesion assessments recognized two dermoscopic characteristics, 20.5% identified three, and 15.4% of the assessments documented four or more. Unfortunately, even though we collected many cases compared to the previously published literature, PM is still a rare tumor, and we are not able to draw statistically significant conclusions from these data regarding the clinical and dermoscopic differences between childhood and teenage melanoma.

## 4. Discussion

Due to its rarity, little is known about PM, and only a few studies have investigated its specific clinical and dermatoscopic features [9,12]. Our retrospective multicenter study provides relevant information concerning the epidemiology and the clinical and dermoscopic presentation of PMs, focusing on their clinical and dermoscopic characteristics. The mean age of diagnosis was 14.6 years (range: 5 to 18 years), and 79.5% of lesions appeared in patients older than 12 years.

A first interesting finding that emerges from our study is the greater incidence of our cases in non-photo-exposed body areas (trunk 39.5% and lower extremities 36.8%) compared to the face and lower limbs (upper extremities,15.8%, head and neck,7.9%), confirming that, given the young ages of our patients, ultraviolet rays in PMs do not represent a risk factor, unlike in adulthood. In this particular age group, family history with melanoma emerges as an important risk factor that we find in more than 25% of cases, compared to the literature figure of about 10% in adult forms [1,18]. These data must always be kept in mind in the management of patients with melanoma who have young children.

Concerning the thickness of the lesions, we found a striking difference in the mean Breslow thickness between the two age groups analyzed (2.02 mm in the 0 to 12 years age group vs. 0.77 mm in adolescents), conforming to the preexisting literature [4].

An analysis of these data indicates this finding is predominantly attributable to female subjects; infact, a significant drop in Breslow thickness can be observed in the over13 age group (from 2.36 mm in the younger children to 0.77 mm in the older subjects).

This cut-off (12 to 13 years) generally corresponds to the onset of menarche in girls and is accompanied by greater hormonal maturity. A possible explanation for this finding could be that the hormonal status may play a role in the pathogenesis of PM. Indeed, many studies have investigated the effect of estrogens and their receptors on melanoma genesis and progression. It is known that estrogen acts through its cognate receptors: estrogen receptor alfa (ERα) and estrogen receptor beta (ERβ) [19,20,21,22]. While ERα has a pro-proliferative effect, ERβ reduces uncontrolled cell proliferation by increasing apoptotic activity [20,21,22,23,24,25]. We believe that these molecular bases can justify our data. Specifically, the effect of estrogens on Erα may be responsible for the increased incidence of melanoma in post-pubertal ages, while their action on ERβ maybe behind the reduction in thickness in the same age range [20,21,22,23,24,25]. However, further studies are needed to confirm this hypothesis, focusing on ER expression in pediatric melanoma tissues and on the actual role of estrogen in neoplastic onset and progression.

Contrary to what has always been described in the literature, from a clinical point of view, about 95% of our cases presented in a pigmented and non-amelanotic form. This datum can perhaps be explained by the phenotypic characteristics of the Mediterranean population enrolled in the study, while the majority of studies in the literature followed an Anglo-Saxon population of Celtic origin with a light phenotype, and must be underlined in the various prevention campaigns where pediatric melanoma is currently associated with a more frequently amelanotic form.

According to the limited literature available, PM often has an atypical clinical presentation that does not follow the typical ABCDEs of melanoma[3,5,9,12],and for this reason, different ABCDE criteria have been proposed in the literature for the early diagnosis of melanoma in childhood[9].To the best of our knowledge, this is the first time that the modified pediatric ABCDE algorithm and the classic ABCDE for clinical diagnosis of melanoma have been compared in a population of patients affected by PM. In contrast to a previous report[9],the majority of our PM cases did not meet the modified ABCDE criteria (Figure 1), being the minority of our PMs amelanotic or presented as bumps or nodules (5.1%), and only 10.5% were a single color. Instead, the classic ABCDE algorithm was the one that best suited our cases (asymmetric: 69.2%, Irregular edges: 64.1%, two or more colors: 59.97%, and over 6 mm in diameter: 56.4%). For these reasons, we believe that the classic ABCDE, despite the limitations already described in the literature, represents a useful algorithm for the non-dermatologist to recognize suspicious lesions early so they can be subjected to a second-level examination with noninvasive diagnostic methods.

Regarding histopathology, the most frequent histological types were superficial spreading melanoma and spitzoid melanoma. In contrast to previous studies [3,5,19,26,27], we did not find any nodular melanoma. However, three rare melanomas were collected (deep penetrating melanoma, nevoid melanoma, and spindle cell melanoma) (Figure 2A–F).

Among the dermoscopic criteria Carrera et al. identified four mainly patterns such as multicomponent/nevus associated, nevus-like with symmetric structures and scant melanoma local features, atypical pink, or pigmented reed spitzoid pattern [12].

The present study indicates that cutaneous dermoscopic parameters of PM are basically the same as in adults (Table 2).

We found that the most common global dermoscopic feature of PM is the presence of irregular streaks/pseudopods (74.36%) (Figure 2B,D). We can also find this dermoscopic parameter in reed nevi, typical of this age group, but in a regular shape and arrangement. Furthermore, in reed nevi, we did not find other dermoscopic parameters typical for suspicious lesions. In PMs, moreover, the irregular streaks/pseudopods were frequently accompanied by different local dermoscopic clues of melanoma, such as irregularly distributed globules, irregular pigmentation, atypical pigment networks, regression structure, and an atypical vascular pattern. Each of these parameters is found in more than 30% of cases. Indeed, all the PMs analyzed presented at least two dermoscopic criteria of melanoma, suggesting that this could be a key for the dermoscopic diagnosis of suspected pediatric melanoma, making it possible to reach an early diagnosis even in this age group.

**Figure 3:**
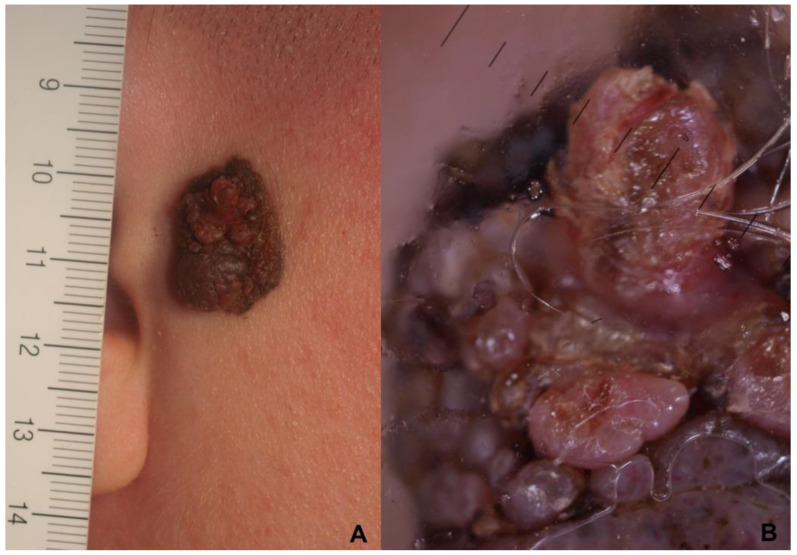
(**A**,**B**) Superficial spreading melanoma developed within a medium-sized congenital melanocytic nevi in a female 18-year-old patient: (**A**) Clinical presentation (ruler is in millimeters): an achromic papillomatous neoformation can be observed in the context of the nevus. (**B**) Dermoscopy atypical and aspecific vascular pattern within the growing achromic lesions.

From the point of view of the clinical outcomes, after an average follow-up of more than eight years (median 76 months), the prognosis seems to be better than for adult melanoma. Out of 39 cases (9 having a thickness greater than 1.0 mm and 4 greater than 2 mm), we found only one disease progression leading to the death of the patient because of a late diagnosis. The patient was the case of an 18-year-old female who developed a 3.6 mm deep melanoma on the preauricular area within a medium-sized congenital melanocytic nevus. Unfortunately, the papillomatous appearance of the congenital pigmented lesion prevents the early clinical and dermoscopic recognition of the change from the congenital nevus to melanoma. Clinically, only an achromic papillomatous neoformation was detectable in the context of the nevus (Figure 3A). Dermoscopy of the lesion showed only an atypical and aspecific vascular pattern within the growing achromic lesions (Figure 3B). Unfortunately, in this case, an impossible early clinical diagnosis resulted in missed opportunities for early intervention, and the tumor had already spread to visceral organs.

In nine cases, LNSB was carried out, and four cases (44.4%) were positive. This percentage is significantly higher than in adults [28,29,30], but only one case subsequently continued to disease progression, in line with the previous literature [4].

Our study has some limitations. First, the retrospective study design. The second limitation is that, although providing important information that can improve the sensitivity for detecting PMs, this study does not address the impact on specificity.

Lastly, although this study is one of the largest to date, the number of lesions included is still too limited to look for other possible predisposing factors for the onset of PM. Thus, larger studies are needed to further corroborate our preliminary findings.

## 5. Conclusions

In conclusion, in contrast to previous reports, the present study highlights that the majority of PMs look similar to adult melanomas, both clinically and dermoscopically. We believe that the use of dermoscopy can complement the clinical examination, providing a helpful supporting tool for the diagnosis of PMs. In our clinical series, contrary to the data published so far, PMs occurred almost always in a pigmented form, and the majority of our PM cases did not meet the modified ABCDE criteria. Conversely, classic ABCDE, despite limitations, already described in the literature, seems to be the most useful algorithm to diagnose PMs early. Namely, dermatologists and pediatricians should remain cognizant that many melanomas in individuals under 18, especially those with a family history and multiple atypical moles, may present exactly as would be expected in young adults.

For this reason, we believe that the joint use of both diagnostic algorithms (classic ABCDE and modified pediatric ABCDE) and dermoscopy represents the most valid diagnostic strategy, guaranteeing greater sensitivity and fewer missed diagnoses. Suspicious lesions deserve further diagnostic investigation by biopsy, since, if diagnosed early, pediatric melanoma has an excellent prognosis.

## Figures and Tables

**Table 1:** Demographics, histopathological parameters, anatomic locations, risk factors, and outcomes in 39 patients under 18 years of age with pediatric melanoma.

Histopathological Parameters	0–12 Years	13–18 Years	Overall
**Ulceration, n (%)**	1/8 (12.5)	1/31 (3.2)	2/39 (5.1)
**Mean Breslow thickness, millimeters, mean (SD)**	2.02 (0.74)	0.77 (0.17)	1.05 (0.23)
**Males**	0.88 (0.51)	0.78 (0.23)	0.80 (0.20)
**Females**	2.36 (1.01)	0.77 (0.25)	1.21 (0.26)
**In situ M, n (%)**	0/8	9/31 (29.0)	9/39 (23.1)
**Males**		3/31 (9.7)	3/39 (7.7)
**Females**		6/31 (19.3)	6/39 (15.4)
**Spitzoid melanoma, n (%)**	7/8 (87.5)	8/31 (25.8)	15 (38.5)
**SSM, n (%)**	0/8	18/31 (58.1)	18/39 (46.2)
**Melanoma arising in congenital melanocytic Nevus, n (%)**	0/8	2/31 (6.5)	2/39 (5.1)
Rare melanoma *, n (%)	1/8 (12.5)	2/31 (6.5)	3/39 (7.8)
**Anatomic location**	**0–12 years**	**13–18 years**	**Overall**
Head/neck, n (%) **	0/7	3/31 (9.7)	3/38(7.9)
Trunk, n (%) **	6/7 (85.7)	9/31 (29.0)	15/38 (39.5)
Upper limbs, n (%) **	0/7	6/31 (19.4)	6/38 (15.8)
Lower limbs, n (%) **	1/7 (14.3)	13/31 (41.9)	14/38 (36.8)
**Risk factors**	**0–12 years**	**13–18 years**	**Overall**
**Multiple primary MM, n (%)**	0/8	3/31 (9.7)	3/39 (7.7)
**Photo-type I, n (%)**	6/8 (75.0)	13/31 (41.9)	19/39 (48.7)
**Familial MM, n (%)**	2/8 (25.0)	8/31 (25.8)	10/39 (25.6)
**Outcome**	**0–12 years**	**13–18 years**	**Overall**
**SNB performed, n (%)**	4/8 (50.0)	5/31 (16.1)	9/39 (23.1)
**Positive SNB, n (%)**	1/8 (12.5)	3/31 (9.7)	4/39 (10.3)
**Metastasis, n (%)**	0/8	1/31 (3.2)	1/39 (2.6)

* Rare melanomas: deep penetrating melanoma, nevoid melanoma, and spindle cells melanoma. ** Parameter not available for the entire study group.

**Table 2:** Dermoscopic features in 39 patients under 18 years of age with pediatric melanoma.

Dermoscopic Features	%
Irregular streaks/pseudopods	74.4
Atypical pigment networks	30.8
Atypical globules	30.8
Regression structures	30.8
Atypical vascular pattern	20.5
Blue white veil	15.4
Inverse network	10.3
Scar-like areas	10.3
Prominent network	10.3
Amelanosis	5.1

## Data Availability

Data available on request due to privacy/ethical restrictions.
